# Effects of ammonium pulse on the growth of three submerged macrophytes

**DOI:** 10.1371/journal.pone.0219161

**Published:** 2019-07-24

**Authors:** Xiaoyao Tan, Guixiang Yuan, Hui Fu, Hui Peng, Dabing Ge, Qian Lou, Jiayou Zhong

**Affiliations:** 1 Hunan Provincial Key Laboratory of Rural Ecosystem Health in Dongting Lake Area, Ecology Department, College of Bioscience and Biotechnology, Hunan Agricultural University, Changsha, China; 2 Jiangxi Provincial Key Laboratory of Water Resources and Environment of Poyang Lake, Jiangxi Institute of Water Sciences, Nanchang, China; Chinese Academy of Sciences, CHINA

## Abstract

Ammonium pulse attributed to runoff of urban surface and agriculture following heavy rain is common in inland aquatic systems and can cause profoundly effects on the growth of macrophytes, especially when combined with low light. In this study, three patterns of NH_4_-N pulse (differing in magnitude and frequency) were applied to examine their effects on the growth of three submersed macrophytes, namely, *Myriophyllum spicatum*, *Potamogeton maackianus*, and *Vallisneria natans*, in terms of biomass, height, branch/ramet number, root length, leaf number, and total branch length under high and low light. Results showed that NH_4_-N pulse caused negative effects on the biomass of the submerged macrphytes even on the 13th day after releasing NH_4_-N pulse. The negative effects on *M*. *spicatum* were significantly greater than that on *V*. *natans* and *P*. *maackianus*. The effects of NH_4_-N pulse on specific species depended on the ammonium loading patterns. The negative effects of NH_4_-N pulse on *P*. *maackianus* were the strongest at high loading with low frequency, and on *V*. *natans* at moderate loading with moderate frequency. For *M*. *spicatum*, no significant differences were found among the three NH_4_-N pulse patterns. Low light availability did not significantly aggregate the negative effects of NH_4_-N pulse on the growth of the submersed macrophytes. Our study contributes to revealing the roles of NH_4_-N pulse on the growth of aquatic plants and its species specific effects on the dynamics of submerged macrophytes in lakes.

## 1. Introduction

In ecosystems, resource supply is rarely constant but varies in frequency and magnitude; therefore, “resource pulse” often occurs as a common ecological phenomenon [1–3]. Resource pulses can affect the physiological metabolism of plants, change their species pattern, and even influence the ecosystem function [4–6]. The effects of resource pulses have been widely explored in terrestrial ecosystems at some ecological scales, such as specific species [7, 8]; wild population [9–11]; community [12–16]; and ecosystem [17, 18]. However, few studies have focused on determining resource pulse effects on aquatic systems [3]. In fact, runoff of urban surface and agriculture following heavy rain can lead to several-fold increases in nutrient concentrations in lake, sewage channel or river [3, 19]. This phenomenon is common in the middle-lower reaches of the Yangtze River in China where many lakes are located, which will largely aggravate lake eutrophication and thus influence the survival and growth of submersed macrophytes [3, 19, 20].

Excess nitrogen in resource pulse following heavy rain will influence the growth and survival of submerged macrophytes [21–27]. In general, submerged macrophytes are more sensitive to ammonium than to nitrate in water column, especially in eutrophic lakes [28]. Free NH_3_ decreases the chlorophyll content [29–31], inhibits respiration, and affects the electron transport system of plants [32]. High concentrations of ammonium will also influence carbon and nitrogen metabolism in submerged plants by reducing carbohydrates, which are consumed as C-skeleton for free amino acid (FAA) synthesis to prevent NH_4_^+^ toxicity, and by increasing FAA [33–36]. Moreover, low light availability aggravates the toxicity of ammonium due to insufficient supply of carbohydrates, leading to degradation of submerged macrophytes [13, 37–39], such as *Potamogeton crispus* [35, 40], *Vallisneria natans* [31], *Vallisneria americana* [41], *Ceratophyllum demersum*, and *Myriophyllum spicatum* [38]. Previous studies on ammonium toxicity were mainly carried out in constant NH_4_-N concentration [20, 40, 42, 43]. Thus, effect of NH_4_-N pulse on the growth of submerged macrophytes needs to be further studied [39].

In this study, three submersed macrophytes, namely, *M*. *spicatum*, *Potamogeton maackianus*, and *V*. *natans*, were used to test the effects of NH_4_-N pulse on their growth. These three species were selected because of the following: (1) they can be found in water from mesotrophic to eutrophic conditions and are widely distributed in the middle-lower reaches of the Yangtze River [44–47]; (2) they have different growth forms, that is, canopy forming for *M*. *spicatum* and rosette forming for *V*. *natans*; (3) *M*. *spicatum* tends to accumulate large amounts of nitrogen under high NH_4_-N condition. *P*. *maackianus* and *V*. *natans* show high ability of maintaining carbon constant [21, 39]; and (4) *V*. *natans* is characterized by low-light compensation point [48, 49].

This study aims to investigate the effects of NH_4_-N pulse on the growth of *M*. *spicatum*, *P*. *maackianus*, and *V*. *natans* under two light treatments. Three patterns of NH_4_-N pulse, differing in magnitude and frequency, were applied to the plants. Responses to NH_4_-N pulse were compared among the three species, and possible mechanisms were discussed. We tested the following hypotheses: (1) ammonium inhibits the growth of plants, and low light availability exacerbates the ammonium effects because of reduced photosynthetic carbohydrate production [34, 35]; (2) ammonium pulse with high loading and low frequency will be more harmful to the growth of submersed macrophytes compared with ammonium pulse with low loading and high frequency because the former causes more severe damage to the cell structure [43]; and (3) *M*. *spicatum* will be more sensitive to NH_4_-N pulse due to its high ability to accumulate nitrogen [21].

## 2. Materials and methods

The experiment was conducted from 7 June to 5 July 2018 in an open space located at Hunan Agricultural University (28°11′N, 113°4′E) in Hunan Province, China. Apical shoots (15 cm length) of *M*. *spicatum* and *P*. *maackianus* and intact plants (10 cm height) of *V*. *natans* were collected in a lake, transplanted into 1152 experimental cups (diameter: 6.0 cm, height: 7.5 cm) containing 6 cm sediment (one apical shoot of *M*. *spicatum* in a cup, two apical shoots of *P*. *maackianus* in a cup, and one plant of *V*. *natans* in a cup). The plants/shoots were placed uniformly in 96 experimental buckets (diameter: 33.0 cm, height: 40.0cm; 12 cups in each bucket; 32 buckets per species). Each bucket contained 35 L of water, and tap water was refilled appropriately to compensate the loss of water due to evaporation every day.

**Fig 1:**
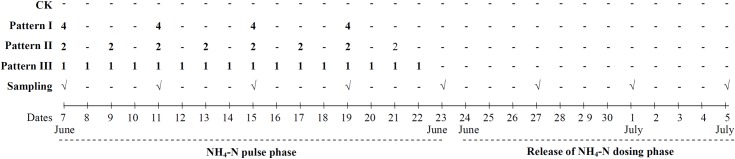
Experimental design. The experiment mainly consisted of NH_4_-N pulse phase and releasing of NH_4_-N dosing phase. During NH_4_-N pulse phase, three NH_4_-N loading patterns were applied to plants, namely, Pattern I - high NH_4_-N (4 mg L^−1^) loading with low pulse frequency; Pattern II - moderate NH_4_-N (2 mg L^−1^) loading with moderate pulse frequency; Pattern III - low NH_4_-N (1 mg L^−1^) loading with high pulse frequency. CK: without NH_4_-N loading. During release of NH_4_-N loading phase, water in the experiment was refreshed with tap water in 23 June. Plant samples were collected in morning every four days.

The experimental design consisted of four replications for two light treatments (HL: high light of about 50% sunlight; LL: low light of about 25% sunlight) and three patterns of NH_4_-N loading for each species. The experiment lasted for 57 days. During plant acclimatization, the plants were incubated in buckets filled with tap water; *M*. *spicatum* and *P*. *maackianus* had developed some roots during this phase, which lasted for 28 days. In the phase of NH_4_-N pulse (Phase I), three patterns of NH_4_-N loading were applied to the plants within 16 days with total NH_4_-N loading of 6551 mg m^-2^ for each pattern. Pattern I [high NH_4_-N loading with low pulse frequency (HL)]: ammonium chloride solution was added to 24 buckets (eight buckets per species) in the morning with NH_4_-N loading of 1637.7 mg m^-2^ every four days for four times (namely 4 mg L^-1^ NH_4_-N each time after conversion according to water volume); Pattern II [moderate NH_4_-N loading with moderate pulse frequency (MM)]: NH_4_-N loading of 818.8 mg m^-2^ every two days for eight times (namely 2 mg L^−1^ NH_4_-N each time after conversion according to water volume) was applied to 24 buckets with eight buckets per species; and Pattern III [low NH_4_-N loading with high pulse frequency (LH)]: NH_4_-N loading of 409.4 mg m^-2^ every day for sixteen times (namely 1 mg L^−1^ NH_4_-N each time after conversion according to water volume) was applied to 24 buckets. Twenty-four buckets (eight buckets per species) without NH_4_-N loading were used as control. In the phase of releasing NH_4_-N dosing (Phase II), water in all of the buckets was refreshed with tap water and the plants were maintained for 13 days. In Phases I and II, one cup of plant in each bucket was collected every four days and growth parameters were measured. 23 June 2018 was the last sampling time of Phase I, and 5 July 2018 was the last sampling time of Phase II, and more detailed descriptions were shown in Fig 1.

During the experiment, water in each bucket was renewed and filamentous algae on the plant leaves were gently removed every 7 days. The water temperature was 29.8°C–36.9°C. The concentrations of TN, TP, NO_3_-N, NH_4_-N, and PO_4_-P in the water column were 0.198 ± 0.079, 0.01 ± 0.018, 0.114 ± 0.036, 0.001 ± 0.004, and 0.0018 ± 0.000 mg L^−1^, respectively, and maintained relatively stable during the experimental period. The concentrations of alkali-hydrolyzable N, available P, available K, and organic material in the sediments were 0.11, 0.03, 0.12, and 19.95 mg g^−1^, respectively. The pH of the sediments was 6.59.

The plant samples were washed with distilled water three times and carefully separated into leaves, stems, and roots for *M*. *spicatum* and *P*. *maackianus* and separated into leaves and roots for *V*. *natans*. The samples oven dried at 80°C to constant weight, and growth parameters were measured and recorded (shoot/plant height, leaf number, ramet number, root length, branch number, and total branch length).

SPSS software was used for statistical analyses. Values were expressed as means ± standard error (SE). All data were tested for normality and homogeneity before analyses. ANOVA was performed to evaluate the effects of NH_4_-N pulse and light treatment on the growth of the three submerged macrophytes. Means were compared by Duncan’s multiple range tests. Explained variances of growth parameters were analyzed using three-way ANOVA, with biomass, height, branch/ramet number, root length, leaf number, and total branch length as dependent variables, with light treatments, NH_4_-N pulse patterns and experimental phases (NH_4_-N pulse phase and release of NH_4_-N loading phase) as fixed factors, and interactions between the three fixed factors considered.

## 3. Results

### 3.1 Effect of NH_4_-N pulse on *M*. *spicatum*

**Fig 2:**
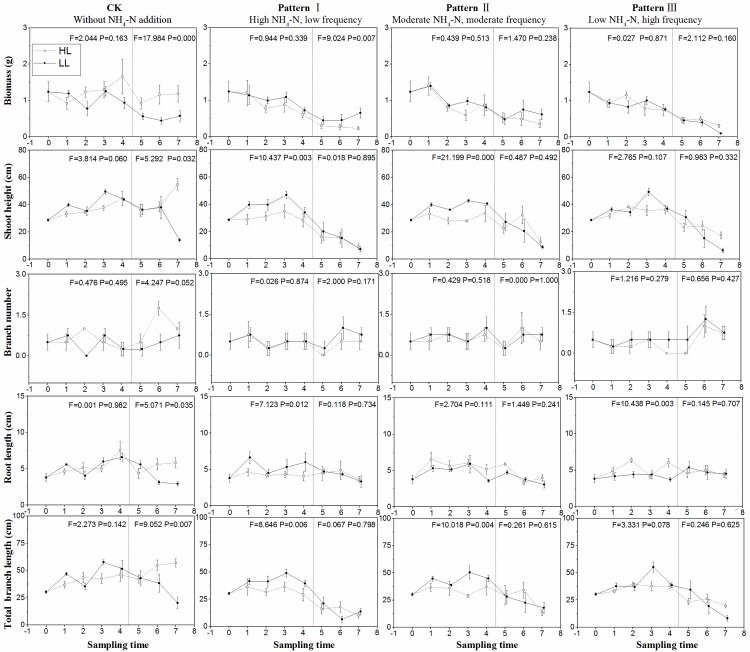
Growth parameters (mean ± SE, *n* = 4) including biomass, height, branch number, root length, and total branch length of *M*. *spicatum* in different sampling times under the high light treatment (○---) and low light treatment (●—) in different patterns of NH_4_-N pulse. Initial values are shown at time 0. Values during NH_4_-N pulse phase and release of NH_4_-N loading phase are shown in the first–fourth time and the fifth–seventh time, respectively. The F and P values of one-way ANOVA between high and low light treatments in the two phases are also shown.

**Fig 3:**
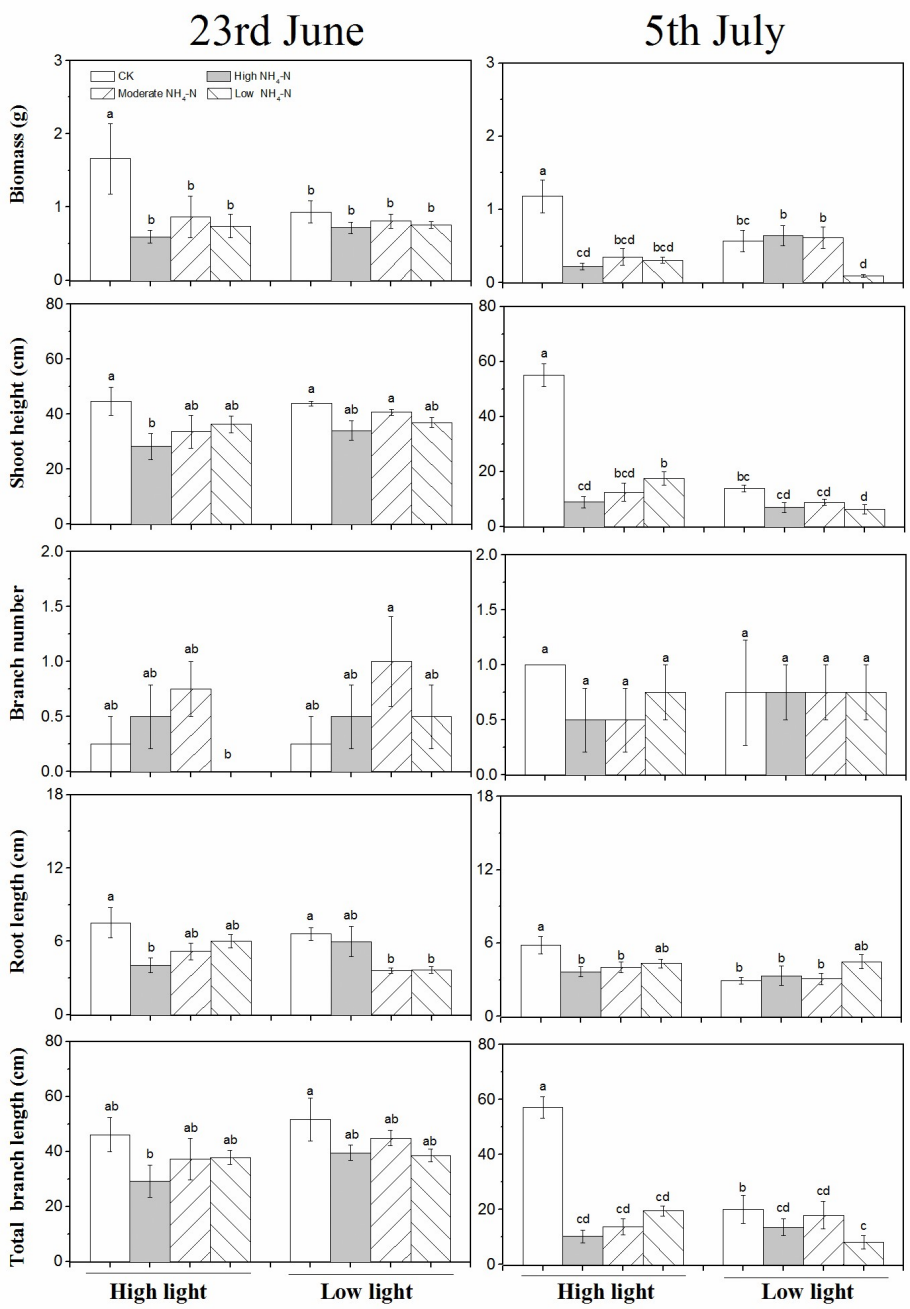
Growth parameters (mean ± SE, *n* = 4) including biomass, height, branch number, root length, and total branch length of *M*. *spicatum* in response to different patterns of NH_4_-N pulse under high and low light on June 23 (the sixteenth day during NH_4_-N pulse phase) and July 5 (the thirteenth day during release of NH_4_-N loading phase).

The NH_4_-N pulse patterns, experimental phases, and interaction of light availability and pulse patterns significantly affected the biomass of *M*. *spicatum* (Table 1, P<0.001). In the control, biomass was higher in high light than in low light during release of NH_4_-N loading phase, while biomass was lower in high light under high NH_4_-N loading with low pulse frequency during this phase (Fig 2, P<0.01). Overall, the three patterns of NH_4_-N pulse gradually decreased the biomass (Fig 2 and Table 1). Biomass in high light was the highest among the eight groups on June 23 and July 5, whereas biomass in low light was the lowest under LH on July 5 (Fig 3).

**Table 1:** Percentage (%) of explained variance based on three-way ANOVA for change in biomass, height, branch number, root length, and total branch length of *M*. *spicatum* in NH_4_-N pulse patterns under high and low light during NH_4_-N pulse and release of NH_4_-N loading phases. Boldface type indicates significant difference at the level of P < 0.05.

		Light (L)	NH_4_-N pulse patterns (N)	Phases (P)	L× N	L ×P	N×P	L×N×P
Biomass	%	0.4	9.5	81.5	6.6	0.3	0.7	0.9
	P	0.467	**0.000**	**0.000**	**0.000**	0.553	0.472	0.359
Height	%	0.2	10.5	71.5	1.2	11.5	4.7	0.4
	P	0.499	**0.000**	**0.000**	0.067	**0.000**	**0.000**	0.536
Branch number	%	1.1	8.4	29.3	28.0	1.3	21.4	10.4
	P	0.742	0.495	0.097	**0.049**	0.724	0.110	0.397
Root length	%	10.5	3.6	55.9	8.8	2.1	7.3	11.8
	P	0.077	0.352	**0.000**	**0.050**	0.422	0.089	**0.015**
Total branch length	%	0.2	15.6	63.6	1.5	12.8	5.6	0.7
	P	0.544	**0.000**	**0.000**	0.063	**0.000**	**0.000**	0.290

The shoot heights of *M*. *spicatum* were markedly influenced by the NH_4_-N pulse patterns, experimental phases, interaction of light availability and experimental phases, and pulse patterns and experimental phases (Table 1, P<0.001). Compared with LH, HL and MM evidently affected the shoot height during NH_4_-N pulse phase, showing higher shoot height in low light than in high light (Fig 2 and Table 1). The shoot height in the control was higher than that in HL on June 23 and was the highest among the eight groups on July 5. The shoot height showed an increasing trend from HL to LH (Fig 3).

The branch number of *M*. *spicatum* was significantly affected by the interaction of light availability and NH_4_-N pulse pattern only (Table 1, P = 0.049) and varied among the eight groups on June 23 and July 5, expect in the group treated with low NH_4_-N loading in high light on June 23 (Fig 2).

The root length was significantly influenced by the experimental phases, interaction of light availability and NH_4_-N pulse patterns, and interaction of the three factors (Table 1). During the NH_4_-N pulse phase, the root length of plants in low light was longer than that of plants in high light under high NH_4_-N loading; by contrast, the root length in low light was shorter than that in high light under low NH_4_-N loading (Fig 2). On June 23 and July 5, the root length under high NH_4_-N loading in high light was significantly shorter than that in the control (Fig 3). Moreover, the root length kept invariable among the patterns of NH_4_-N pulse, except for the control (Fig 3).

The total branch length of *M*. *spicatum* was markedly influenced by NH_4_-N pulse patterns, experimental phases, interaction of light availability and experimental phases, and pulse patterns and experimental phases (Table 1, P<0.001). The total branch length in low light was longer than that in high light under high and moderate NH_4_-N loading (Fig 2). Similar to shoot height, the total branch length showed an increasing trend from high to low NH_4_-N loading in high light (Fig 3).

### 3.2 Effects of NH_4_-N pulse on *P*. *maackianus*

**Fig 4:**
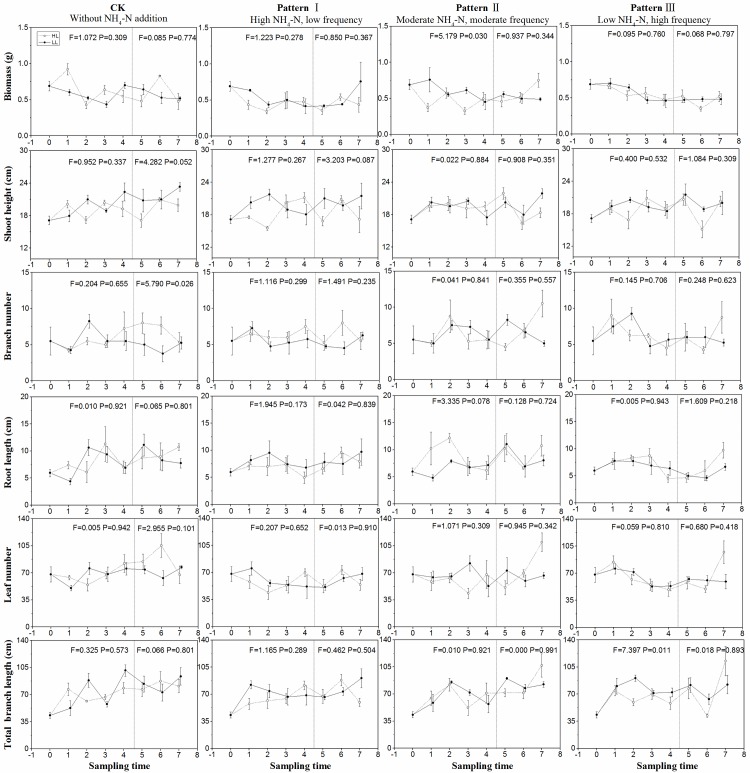
Growth parameters (mean ± SE, *n* = 4) including biomass, height, branch number, root length, leaf number, and total branch number of *P*. *maackianus* in different sampling times under the high light treatment (○---) and low light treatment (●—) in different patterns of NH_4_-N pulse. Initial values are shown at time 0. Values during NH_4_-N pulse phase and release of NH_4_-N loading phase are shown in the first–fourth time and the fifth–seventh time, respectively. The F and P values of one-way ANOVA between high and low light treatments in the two phases are also shown.

**Fig 5:**
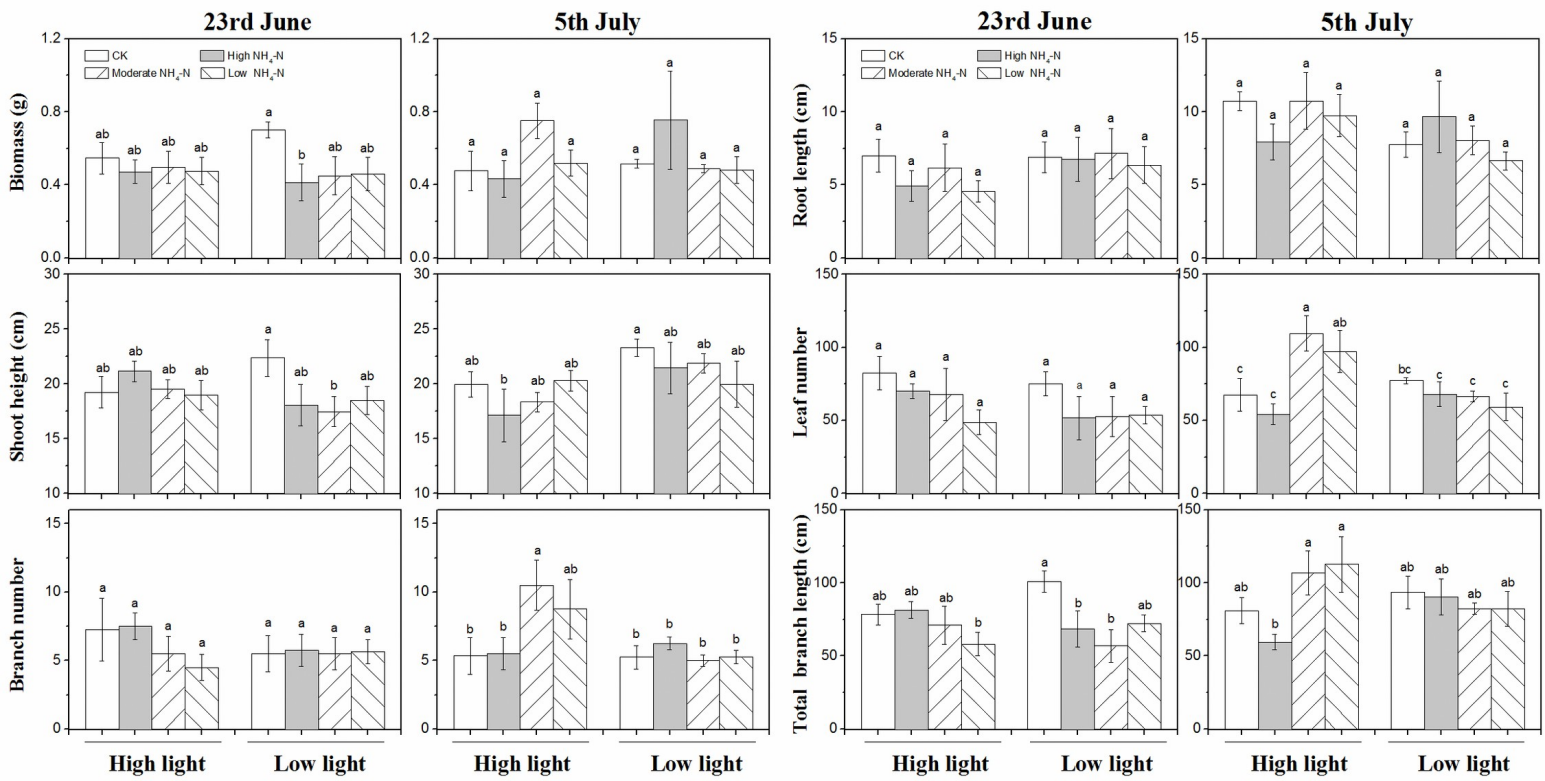
Growth parameters (mean ± SE, *n* = 4) including biomass, height, branch number, root length, leaf number, and total branch number of *P*. *maackianus* in response to different patterns of NH_4_-N pulse under high and low light on June 23 (the sixteenth day during NH_4_-N pulse phase) and July 5 (the thirteenth day during release of NH_4_-N loading phase).

NH_4_-N pulse patterns significantly affected the biomass of *P*. *maackianus* (Table 2, P<0.05). Under moderate NH_4_-N loading, the biomass was higher in low light than that in high light during NH_4_-N pulse phase (Fig 4, P<0.05). The biomass in the control was higher than that under high NH_4_-N loading in low light on June 23, while the biomass in the other groups kept invariable (Fig 5).

**Table 2:** Percentage (%) of explained variance based on three-way ANOVA for change in biomass, height, branch number, root length, leaf number, and total branch number of *P*. *maackianus* treated with NH_4_-N pulse patterns under high and low light during NH_4_-N pulse phase and release of NH_4_-N loading phase. Boldface type indicates significant difference at the level of P < 0.05.

		Light (L)	NH_4_-N pulse patterns (N)	Phases (P)	L× N	L ×P	N×P	L×N×P
Biomass	%	10.0	35.2	5.9	12.0	5.2	15.2	16.5
	P	0.322	**0.018**	0.446	0.321	0.475	0.219	0.187
Height	%	67.1	4.8	4.3	4	18.2	1.1	0.3
	P	**0.001**	0.511	0.404	0.585	0.088	0.907	0.982
Branch number	%	30.8	12.7	0.2	5.1	35.8	8.7	6.7
	P	0.091	0.317	0.903	0.700	0.069	0.487	0.598
Root length	%	7.8	38	17.2	11.7	0.7	18.2	6.4
	P	0.379	**0.011**	0.192	0.324	0.785	0.145	0.593
Leaf number	%	4.4	28.2	31.1	2.8	25.6	5.3	2.6
	P	0.397	**0.004**	**0.025**	0.716	**0.042**	0.460	0.737
Total branch length	%	13.2	5.2	65.6	1.6	5.3	6.3	2.8
	P	0.163	0.513	**0.002**	0.871	0.376	0.428	0.745

The shoot height was significantly affected by light availability (Table 2, P<0.05). The shoot height was longer in the control than under moderate NH_4_-N loading in low light on June 23 and was not significantly different among the other groups (Fig 5).

In the control, the branch number in high light was significantly higher than that in low light during the release of loading phase (Fig 3, F = 5.790 P = 0.026). On July 5, the plants showed higher branch number under moderate NH_4_-N loading than under control and high NH_4_-N loading (Fig 5).

The root length was marginally higher in high light than in low light under moderate NH_4_-N loading during the release of loading phase. The leaf number was markedly affected by the NH_4_-N pulse patterns, experimental phases, and interaction of light and experimental phases (Table 2). On July 5, the plants showed greater leaf numbers under moderate and low NH_4_-N loading than under control and high NH_4_-N loading; meanwhile, the leaf number did not change among the other groups (Fig 5).

The total branch length was affected by experimental phases (Table 2). On June 23, the total branch length was longer under control than under high and moderate NH_4_-N loading. On July 5, the total branch length under moderate and low NH_4_-N loading was significantly greater than those under high NH_4_-N loading (Fig 5).

### 3.3 Effects of NH_4_-N pulse on *V*. *natans*

**Fig 6:**
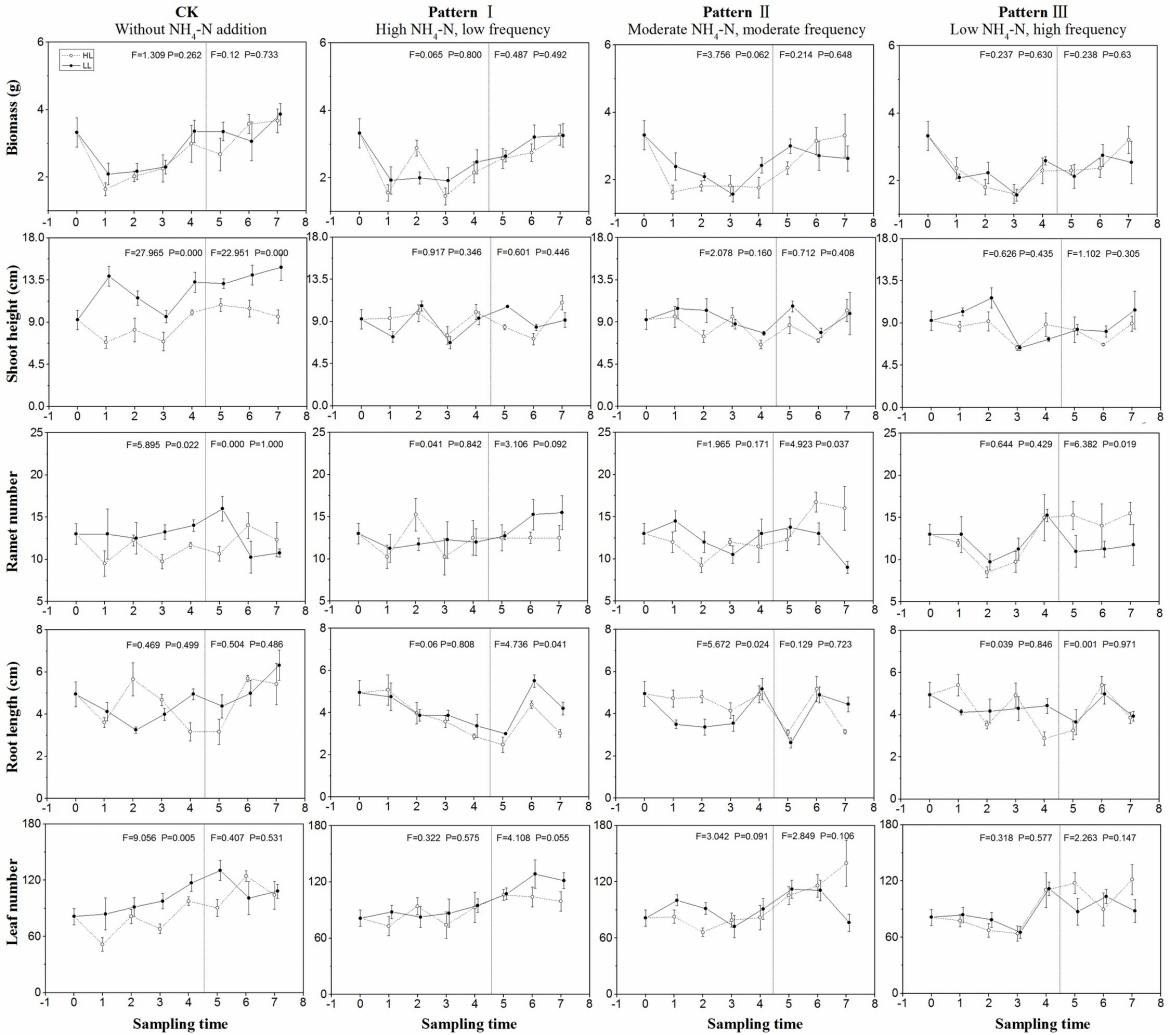
Growth parameters (mean ± SE, *n* = 4) including biomass, height, ramet number, root length, and leaf number of *V*. *natans* in different sampling times under the high light treatment (○---) and low light treatment (●—) in different patterns of NH_4_-N pulse. Initial values are showed at time 0. Values during NH_4_-N pulse phase and release of NH_4_-N loading phase are showed in the first–fourth time and the fifth–seventh time, respectively. The F and P values of one-way ANOVA between high and low light treatments in the two phases are also shown.

**Fig 7:**
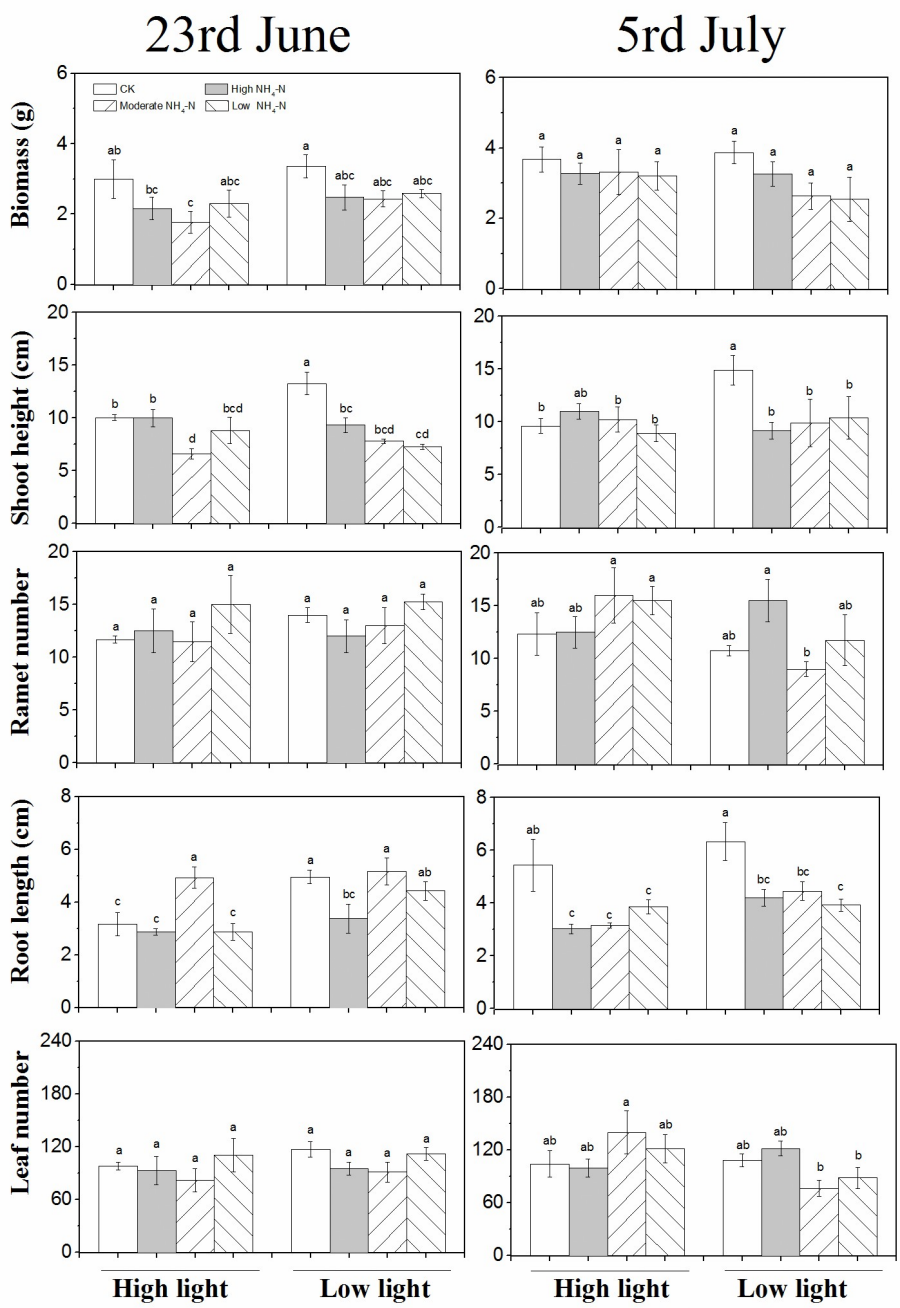
Growth parameters (mean ± SE, *n* = 4) including biomass, height, ramet number, root length, and leaf number of *V*. *natans* in response to different patterns of NH_4_-N pulse under high and low light on June 23 (the sixteenth day during NH_4_-N pulse phase) and July 5 (the thirteenth day during release of NH_4_-N loading phase).

The biomass of *V*. *natans* was markedly affected by NH_4_-N pulse patterns and experimental phase (Table 3). During pulse phase, the biomass in low light was marginally greater than that in high light under moderate NH_4_-N loading (Fig 6). The biomass was significantly lower under moderate NH_4_-N loading than under control on June 23 but did not change among all the eight groups on July 5 (Fig 7).

**Table 3:** Percentage (%) of explained variance based on three-way ANOVA for change in biomass, height, ramet number, root length, and leaf number of *V*. *natans* in NH_4_-N pulse patterns under high and low light during NH_4_-N pulse phase and release of NH_4_-N loading phase. Boldface type indicates significant difference at the level of P < 0.05.

		Light (L)	NH_4_-N pulse patterns (N)	Phases (P)	L× N	L ×P	N×P	L×N×P
Biomass	%	1.3	6.7	87.9	0.3	1.4	1.9	0.5
	P	0.282	**0.001**	**0.000**	0.848	0.267	0.169	0.699
Height	%	42.0	26.9	8.9	15.8	0.2	5.5	0.7
	P	**0.000**	**0.000**	**0.025**	**0.000**	0.707	**0.026**	0.736
Ramet number	%	0.0	1.6	37.1	9.8	33.1	2.0	16.3
	P	0.966	0.804	**0.006**	0.109	**0.009**	0.743	**0.019**
Root length	%	2.9	32.1	0.9	9.2	30.6	20.1	4.3
	P	0.537	**0.006**	0.728	0.307	**0.046**	**0.050**	0.636
Leaf number	%	2.1	1.3	81.1	4.0	7.9	0.4	3.1
	P	0.235	0.463	**0.000**	**0.046**	**0.023**	0.832	0.100

The shoot height was significantly influenced by light availability, NH_4_-N pulse patterns, experimental phases, interactions of light and pulse patterns, and interactions of pulse patterns and experimental phases (Table 3, P < 0.05). The shoot height differed among treatments with varying light availability during both phases under control but was kept unchanging under the three other NH_4_-N pulse patterns (Fig 6). On June 23, the shoot height under moderate NH_4_-N loading was lower than that under control and high NH_4_-N loading. On July 5, the shoot height in low light under control was higher than that under the three other NH_4_-N pulse patterns (Fig 7).

The ramet number showed significant responses to experimental phases, interaction of light and experimental phases, and interaction of the three factors (i.e., light, NH_4_-N pulse patterns, experimental phases). Under control, the ramet number was greater in low light than in high light during pulse phase. Under moderate and low NH_4_-N loading, the ramet number was greater in high light than in low light during the release of loading phase (Fig 6). On July 5, the ramet number was greater under high NH_4_-N loading than under moderate NH_4_-N loading (Fig 7).

The root length was significantly influenced by NH_4_-N pulse patterns and interaction of experimental phases, light, and pulse patterns. Under moderate NH_4_-N loading, the root length was greater in high light than in low light during the pulse phase. On June 23, the root length was the greatest under moderate NH_4_-N loading in high and low light and under control in low light. Meanwhile, the root length was the greatest in control in high and low light (Fig 7).

The leaf number was markedly affected by experimental phases and interactions of light, NH_4_-N pulse pattern, and experimental phase (Table 3). The leaf number was marginally higher in low light than in high light under control and moderate NH_4_-N loading during the pulse phase and under high NH_4_-N loading during the release of loading phase (Fig 6). The leaf number did not change, regardless of NH_4_-N pulse patterns in different light availability levels or experimental phases (Fig 7).

## 4. Discussion

Our experimental results showed that NH_4_-N pulse inhibited the growth of submerged plants by reducing the biomass, height and total branch length on July 5 and the root length under high NH_4_-N loading pulse in high light availability for *M*. *spicatum* and by decreasing the biomass under moderate NH_4_-N loading pulse in high light for *V*. *natans*. This finding is partly consistent with our first hypothesis. In general, excess NH_4_-N would cause stress to submersed macrophytes, and NH_4_^+^ toxicity arising from eutrophication probably plays an important role in the degradation of the plants [20, 21, 29, 50–52]. For example, Cao et al. (2007) set 0.56 mg L^−1^ NH_4_-N in the water column as the upper limit for survival of *V*. *natans* in lakes in the middle-lower reaches of the Yangtze River in China [26]. In the present study, the biomass of *V*. *natans* kept invariable under NH_4_-N concentration of 0–1 mg L^−1^ regardless of light availability. This discrepancy could be attributed to multiple stresses from biotic and abiotic competition, herbivory, and wave exposure, except for NH_4_-N stress and low light availability in field lakes [53, 54]. In our previous study, the biomass of *M*. *spicatum* was constant under stable NH_4_-N concentration of 2 mg L^−1^ for 4 days [55]; by contrast, in the present study, the biomass of *M*. *spicatum* declined obviously under NH_4_-N pulse of 2 mg L^−1^ treated for eight times within 16 days. These findings may suggest that NH_4_-N stress was partly time dependent. Short-term ammonium loading may not inhibit the growth of submersed macrophytes, but long-term exposure could significantly affect the growth of plants.

The effects of NH_4_-N pulse on plants differed among the three pulse patterns and were species specific. For *P*. *maackianus*, the branch number, leaf number, and total branch length were the lowest in high light on July 5 under high NH_4_-N loading pulse. No significant differences in these morphological traits were observed between moderate and low NH_4_-N loading pulses. These results are consistent with our second hypothesis stating the high tolerance of this species to NH_4_-N stress because of its relatively high conservative carbohydrate metabolism [39]. However, high NH_4_-N concentration may decrease the chlorophyll content and affect the electron transport system of plants [30–32], probably explaining the decline in some of the morphological traits for this species. For *V*. *natans*, the ramet number in low light on July 5 and the shoot height in high light on June 23 were the lowest under moderate NH_4_-N loading pulse. The high carbon supply for its low light saturation point may lead to the high turnover of carbon metabolism [56, 57], leading to high rehabilitation capacity, which may benefit the growth of *V*. *natans* exposed to ammonium toxicity of high NH_4_-N loading pulse in the present study. For *M*. *spicatum*, no significant differences or consistent patterns were found among the three NH_4_-N pulse patterns, except for the shoot height in high light on July 5. The carbon supply may be used for maintenance in addition to detoxification in accordance with its high sensitivity to NH_4_-N pulse and high light saturation point [21, 39, 49].

The effects of NH_4_-N pulse on the growth of submersed macrophytes were species specific. In the present experiment, the effect of NH_4_-N pulse on the biomass of *M*. *spicatum* was significantly greater than those on *V*. *natans* and *P*. *maackianus*, conforming to our third hypothesis. *M*. *spicatum* showed a characteristic of acquisitive strategy with relatively high NH_4_-N absorption and growth rates [21, 36, 39, 58]. Large amount of nitrogen could be accumulated in *M*. *spicatum* during NH_4_-N pulse phase, resulting in profound effect on their growth for a long time [21, 59] and inhibited growth due to shortage of carbohydrate consumed as C-skeleton for FAA synthesis. For *V*. *natans*, the carbon supply is relatively sufficient for its low light saturation point [56, 57], suggesting its high ammonium detoxification in the present study. Yuan et al. (2013) found that *V*. *natans* was more efficient at maintaining C-N metabolic homeostasis when faced with acute NH_4_^+^ dosing [39]. For *P*. *maackianus*, the biomass kept unchanged regardless of NH_4_-N pulse patterns and light availability, except for that in high NH_4_-N loading pulse in low light on June 23. Imamoto et al. (2007) reported that *P*. *maackianus* showed positive RGR at only 13% transmittance [60]. This finding may be ascribed to the considerably low light requirements of this species [61]. Previous studies pointed out that *P*. *maackianus* can avoid instant ammonium toxicity by restricting photo-synthesis through uncoupling photophosphorylation, leading to low accumulation of nitrogen even in high levels of NH_4_-N [62].

Low light is a major factor determining the growth of submerged macrophytes [63, 64], which could aggravate the effects of ammonium stress, especially in eutrophic lakes [26]. However, compared with high light in the present study, the biomass was higher in low light for *M*. *spicatum* during the release of loading phase under high NH_4_-N loading and for *P*. *maackianus* and *V*. *natans* during the pulse phase under moderate NH_4_-N loading. This finding partly contradicts our first hypothesis that low light availability exacerbates the ammonium effects. Cao et al. (2011) found that growth of *M*. *spicatum* would not be affected negatively by NH_4_-N under high light but decreased significantly under low light treatments [38]. Similar results were reported for *P*. *crispus* [35, 40], *Potamogeton amplifolius* and *Nuphar advena* [34]. Under high light conditions, plants may show a consumptive carbon strategy, that is, high respiration rate [65, 66], because of sufficient supply of photosynthetic carbon. Apart from maintaining growth, large amount of carbon may be used for ammonium detoxification [21]. By contrast, under low light conditions, plants may adopt conservative carbon strategy when facing carbon starvation by having low respiration and high carbohydrate storage [66–68]. These phenomena could partly promote the results in the present study.

Macropyhte species can modify the morphological traits to overcome environmental and resource stresses [69, 70]. In the present study, the shoot height and total branch length were higher in low light than in high light for *M*. *spicatum* during the pulse phase under high and moderate NH_4_-N loading; moreover, the shoot height and leaf number were greater for *V*. *natans* under control. These results are consistent with previous findings that terrestrial and aquatic plants would allocate more resources to the above ground part by increasing their height or number of leaves to obtain more light and improve their light competitiveness and survival fitness [71–74]. The roots act as organs for absorption and assimilation of nitrogen and phosphorus from sediments particularly in oligotrophic lakes [75, 76]. In the present study, the root length of plants was inhibited under NH_4_-N pulse for *M*. *spicatum* and *V*. *natans*, consistent with previous results of decreasing allocation of underground parts under high nutrients for terrestrial plants [77, 78]. *M*. *spicatum* absorbed NH_4_^+^ mainly by the leaf rather than by the root when NH_4_-N in the water column was about 0.1 mg L^−1^ [75]. These results confirm that the role of the roots as a nutritive organ weakened in the hypertrophic environment [79].

To conclude, NH_4_-N pulse affected the growth of submerged macropytes in species specific and light dependent manner. The negative effects on *M*. *spicatum* were significantly greater than that on *V*. *natans* and *P*. *maackianus*. The negative effects of NH_4_-N pulse on *P*. *maackianus* were the strongest at high loading with low frequency, and on *V*. *natans* at moderate loading with moderate frequency. For *M*. *spicatum*, no significant differences were found among the three NH_4_-N pulse patterns. Low light availability did not significantly aggregate the negative effects of NH_4_-N pulse on the growth of the submersed macrophytes.

## Supporting information

S1 File Supporting information File 1. (XLSX)
